# Infant Mortality

**Published:** 1913-03

**Authors:** 


Infant Mortality. In the United States almost exactly one child in eight dies within the first year. In New York City 16 per cent. die. The lowest mortality for the first year of life is in New Zealand, 68.1000.

New York City has followed the example of Buffalo in supplying free anti-typhoid vaccination, beginning January 1.

